# Mixed Metal Amide-Hydride Solid Solutions for Potential Energy Storage Applications

**DOI:** 10.1021/acs.inorgchem.4c01016

**Published:** 2024-05-30

**Authors:** Thi Thu Le, Simone Bordignon, Michele R. Chierotti, Yuanyuan Shang, Alexander Schökel, Thomas Klassen, Claudio Pistidda

**Affiliations:** † Institute of Hydrogen Technology, Helmholtz-Zentrum hereon GmbH, Max-Planck-Straße 1, Geesthacht D-21502, Germany; ‡ Department of Chemistry, University of Torino, V. P. Giuria 7, Torino I-10125, Italy; § Deutsches Elektronen-Synchrotron DESY, Notkestraße 85, Hamburg D-22607, Germany; ∥ Helmut Schmidt University, Holstenhofweg 85, Hamburg D-22043, Germany

## Abstract

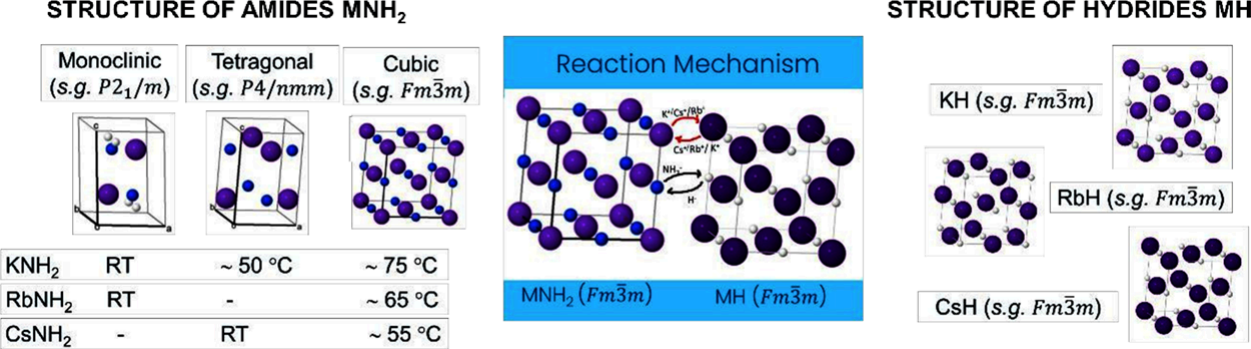

Mixed solid solutions have played an important role in improving the kinetics and performance of hydrogen storage materials, as reported for the Li–Mg–N–H, K–Mg–N–H, and Rb–Mg–N–H systems. Besides, the formation of a homogeneous solid solution, mostly due to partial ionic substitution, is known to be an effective approach to improve the ionic conductivity of a material, which is an important property in electrochemical applications. We have reported a series of solid solutions based on mixed amide-hydride materials of the Group 1 elements, e.g., K(NH_2_)_*x*_H_1–*x*_, Rb(NH_2_)_*x*_H_1–*x*_, and Cs(NH_2_)_*x*_H_1–*x*_, via the exchange of NH_2_^–^/H^–^ anions with the change of the lattice cell of the solid solution. Extending the research in this direction, we study the M–N–H solid solution in the MNH_2_–MH systems (M = K, Rb, Cs, and their combinations), i.e., KNH_2_–RbH, RbNH_2_–KH, RbNH_2_–CsH, and CsNH_2_–RbH via ex situ/in situ XRD, IR, and ^1^H 2D solid-state NMR. The results obtained confirm the formation of mixed metal amide-hydride solid solutions associated with an exchange between both anionic (NH_2_^–^ and H^–^) and cationic species (K^+^, Rb^+^, and Cs^+^). With this study, we aim to create an accessible library of M–N–H solid solutions for further studies as additives for hydrogen storage materials or ionic conductors.

## Abstract 2

Structural and chemical similarities between alkali metal (K, Rb, and Cs) amide and hydride compounds lead to the formation of mixed metal solid solutions that can be further investigated for hydrogen storage and ionic conduction.

## 1 Introduction

Metal amide-hydride materials have been extensively investigated in recent years for use in energy storage applications and specifically for storage applications in hydrogen technology (e.g., LiNH_2_-LiH,^1−6^ Mg(NH_2_)_2_-2LiH,^7−14^ Mg(NH_2_)_2_-KH,^15^ and Mg(NH_2_)_2_-RbH)^16^) and as solid electrolytes^17−19^ for all solid-state batteries. In hydrogen storage applications, amide-hydride systems prove promising candidates especially due to their high hydrogen storage capacity and tunable thermodynamics, which allows hydrogen desorption/absorption to occur at temperatures below 150 °C.^20^ However, the sluggish desorption/absorption kinetics of these amide-hydride systems limit their potential employment in commercial applications. In such cases, metal hydrides-based additives such as KH, RbH, and CsH can effectively improve the hydrogen sorption kinetics and alter the thermodynamics of amide-hydride systems, in particular of Mg(NH_2_)_2_-2LiH.^21−25^ These metal hydrides promote the formation of intermediate phases/solid solutions, which reduce the kinetic barrier of the amide/hydride interfacial reaction and lower the operating temperature of the Li–Mg–N–H system. Other solid solutions, such as the Li–N–H ones (e.g., Li_2+*x*_(NH)_1–*x*_N_*x*_H_*x*_ and Li_2+*x*_NH), were synthesized to modify the thermal stability and ammonia reactivity of the LiNH-LiH system,^6^ while Ca_2_NH–Ca_2_N_2_ solid solutions were studied as catalysts to promote low-temperature ammonia synthesis.^26^ In general, solid solutions are not only important for hydrogen storage but also for the ionic conduction of complex metal hydrides. The formation of a homogeneous solid solution by ion mixing is one of the most common ways to improve the ionic conductivity of a material,^27^ most likely due to the structural perturbations or modifications that facilitate the migration of ions, as observed in the LiBH_4_–LiX (X = Cl^–^, Br^–^, and I^–^)^28,29^ and NaBH_4_–NaI.^30^

**Figure 1:**
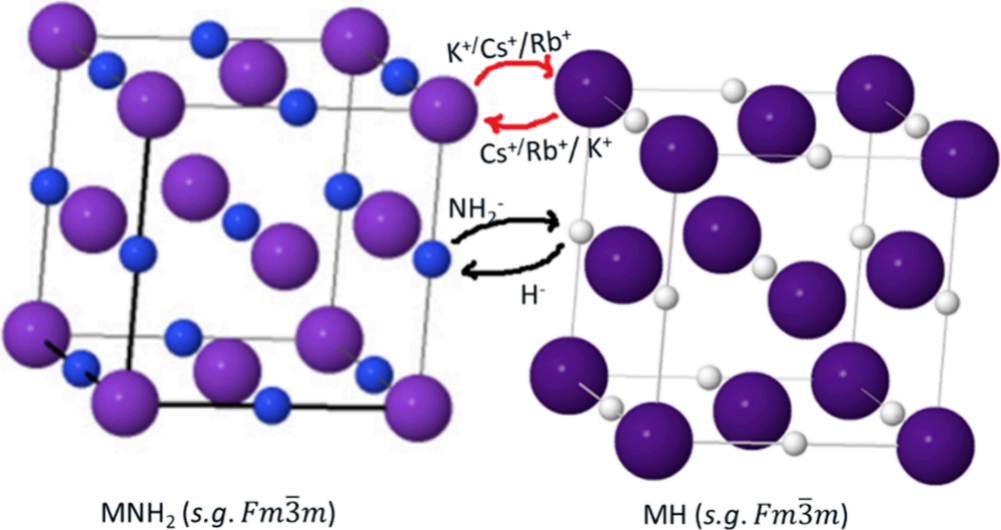
Schematic diagram of the amide-hydride reaction mechanism.

Our previous studies reported the formation of mixed amide-hydride solid solutions, such as K(NH_2_)_*x*_H_1–*x*_, Rb(NH_2_)_*x*_H_1–*x*_, and Cs(NH_2_)_*x*_H_1–*x*_, via the exchange of anionic species (NH_2_^–^ in amides and H^–^ in hydrides) for the KNH_2_–KH,^31^ RbNH_2_–RbH,^16^ and CsNH_2_–CsH^32^ systems using both experimental determinations and theoretical calculations. The relative amides (i.e., KNH_2_, RbNH_2_, and CsNH_2_) undergo a structural transition from monoclinic space group (s.g.) *P*2_1_/*m* (for both KNH_2_ and RbNH_2_) and tetragonal s.g. *P*4/*nmm* (for CsNH_2_) to cubic crystal structures with the same s.g. *Fm*3̅*m* within the temperature range of 50–80 °C. On the contrary, the corresponding hydrides, namely, KH, RbH, and CsH, have similar cubic crystal structures (s.g. *Fm*3̅*m*), which remain unchanged upon temperature variation. Furthermore, the dissolution of NH_2_^–^ into the KH (or RbH and CsH) hydride structure leads to the structural expansion of the M(NH_2_)_*x*_H_1–*x*_ solid solutions (M = K, Rb, and Cs). This change in the lattice structure could lead to significant changes in the material’s functional properties, such as ionic conductivity, similarly to what observed in ionic conducting systems like LiBH_4_–LiX (X = Cl^–^, Br^–^, and I^–^)^28,29^ and NaBH_4_–NaI.^30^ In particular, KNH_2_ exhibits an ionic conductivity of 3.56 × 10^–4^ S cm^–1^ at 150 °C, which can be further enhanced by introducing structural disorder.^18^ These findings suggest a high potential for the application of these amide-hydride solid solutions (with modified structures) in the energy storage field. In this work, we report the formation of solid solutions in the MNH_2_–MH system, namely, KNH_2_–RbH, RbNH_2_–KH, CsNH_2_–RbH, and RbNH_2_–CsH, via the exchange of anion and cation species. It is well-known that several factors determine the limits of solubility. These are expressed as a series of rules, the so-called William Hume–Rothery Rules: (i) atomic size factor (extensive substitutional solid solution occurs only if the relative difference between the atomic radii of the two species is less than 15%); (ii) crystal structures of the two elements must be identical; (iii) two species (the solute and the solvent atoms) should typically have the same valence; and (iv) electronegativity difference was close to 0. In the present study, there are clear structural analogies among these MNH_2_ amides and between them and the MH hydrides. Moreover, comparing the relative difference between the size of K^+^ (*r*_ion_ = 1.52 Å), Rb^+^ (*r*_ion_ = 1.66 Å), and Cs^+^ (*r*_ion_= 1.81 Å) cations and between the size of NH_2_^–^ (*r*_ion_ = 1.73 Å) and H^–^ (*r*_ion_ = 1.53 Å) anions, the radii difference for any of their combinations is less than 15%, which, according to the Hume–Rothery rules, allows for the formation of M(NH_2_)_*x*_H_1–*x*_ solid solutions, based on the substitution of both anion and cation species. The proposed diagram for ion substitutions in these systems is illustrated in Figure 1. As mentioned above, ionic hydrides (KH, RbH, and CsH) have been shown to be effective additives to improve the kinetic and thermodynamic properties of the most promising amide-hydride hydrogen storage system, Mg(NH_2_)_2_-2LiH. Therefore, the use of such mixed metal amide-hydride as additives in the hydrogen storage systems might be possible. Moreover, the formation of solid solutions displaying crystal lattice disorder (due to the different ionic radius of exchanged cations and anions) could create channels for ion diffusion and therefore increase the ionic conduction within the solid lattice, highlighting the potential use of these alkali metal mixed amide-hydride solid solutions for further studies on the ionic conductivity or as dopants/catalysts for hydrogen storage materials.

## 2 Experimental Method

### 2.1 Materials Preparation

Potassium hydride (KH) in paraffin was purchased commercially (35.8 wt % KH purity, Sigma-Aldrich). The synthesis of potassium amide (KNH_2_), rubidium amide (RbNH_2_), rubidium hydride (RbH), cesium hydride (CsH), and cesium amide (CsNH_2_) followed the same procedures, as reported in refs (16 and 31). KNH_2_ was synthesized by repeated ball-milling of the potassium hydride in a Pulverisette planetary mill at 500 rpm with a ball-to-powder ratio (BPR) of 20:1 under 7 bar of NH_3_ for 5 h. This process was repeated four times for a total milling time of 20 h. Between each repetition, the high-pressure vessel was evacuated and refilled with fresh NH_3_. RbH was synthesized by ball-milling metallic rubidium (Rb 99.8%, Alfa Aesar) in 50 bar of H_2_ at 500 rpm for 13 h with a BPR of 60:1, followed by further annealing at 180 °C for 5 h under 70 bar of H_2_. RbNH_2_ was synthesized by heat treatment of metallic Rb in 7 bar of NH_3_ at 250 °C for 13 h. The mixtures *x*KNH_2_ + (1 – *x*)RbH and *x*RbNH_2_ + (1 – *x*)KH with *x* = {0, 0.5, 0.7, 1} were prepared by hand-grinding using an agate mortar followed by heat treatment at 270 °C for 3 h. To avoid oxidation of the materials, all samples were prepared under a protective atmosphere in a continuous Ar-filled glovebox (MBraun, Germany) with an oxygen and humidity concentration of less than 1 ppm.

### 2.2 Materials Characterization

Ex situ powder X-ray diffraction (XRD) experiments were performed using a D8 Discover diffractometer (Bruker AXS GmbH, Karlsruhe, Germany) equipped with a Cu Kα beam (λ = 1.54184 Å) and 2D VANTEC detector. The diffractograms were acquired in the 2θ range from 10° to 90°, in nine steps with an exposure time of 400 s per step. A small amount of the sample was placed on a flat commercial sample holder and sealed with an airtight poly(methyl methacrylate) lid to prevent oxidation.

In situ synchrotron powder X-ray diffraction (in situ SR-PXD) measurements were performed at the Powder Diffraction and Total Scattering Beamline (P02.1) of Petra III (Desy Hamburg, Germany)^33^ using a monochromatic X-ray beam (λ = 0.20734 Å). The diffraction patterns were collected by a Varex4343CT detector with an array of 2880 × 2880 pixels and a pixel size of 150 μm × 150 μm with an exposure time of 10 s per pattern. Samples were loaded into sapphire capillaries under a purified Ar atmosphere and then mounted on an in-house developed in situ cell, in which operating temperatures and pressures can be controlled.^34,35^ The measurements for all mixtures were carried out under 1 bar of Ar, with the sample heated up from room temperature (RT) to 270 °C at a heating rate of 5–10 °C/min, held isothermally at 270 °C for 30 min, and then cooled down to RT. A small difference in the measurement conditions of the starting materials (KNH_2_, RbH, RbNH_2_, and KH) is that they were kept isothermally at 270 °C for 10 min instead of 30 min. The 2D diffraction images were integrated into 1D diffractograms using the Fit2d software, and the quantitative analyses were performed using the Rietveld refinement method with the Material Analysis Using Diffraction software (MAUD).^36^ Structural information on known phases was obtained from the International Crystal Structure Database (ICSD) using the ICSD-Desktop software.

The pure and mixed samples were characterized using the Fourier transform infrared spectroscopy (Cary 630 FT-IR spectrometer, Agilent Technologies Deutschland GmbH, Waldbronn, Germany). The FT-IR spectrometer was placed in an Ar-circulated glovebox with oxygen and moisture concentrations below 5 ppm. The background was calibrated for each measurement; a small amount of material was placed on the diamond ATR top plate, and the FT-IR spectrum was acquired at RT in a full frequency range of 4000–650 cm^–1^ with a spectral resolution of 4 cm^–1^ and a number of scans of 300.

Solid-state nuclear magnetic resonance (SSNMR) experiments were run on a Jeol ECZR 600 instrument, operating at a frequency of 600.13 MHz for ^1^H and equipped with a 3.2 mm probe. Rotors were packed inside a glovebox to prevent sample decomposition. The ^1^H MAS spectra were acquired at probe temperature at a spinning speed of 20 kHz (4 scans; optimized relaxation delays equal to 200 or 280 s, corresponding to 5·T_1_ for quantitative measurements). The 2D ^1^H double-quantum (DQ) MAS experiments were performed at probe temperature at a spinning speed of 20 kHz with the back-to-back (BABA)-xy16 recoupling pulse sequence with excitation time durations of eight rotor periods (^1^H 90° = 2.2 μs; 4 scans; *t*1 increments = 64; relaxation delay = 50 or 72 s, corresponding to 1.27·T_1_). The ^1^H chemical shift scale was calibrated with adamantane (^1^H signal at 1.87 ppm with respect to primary standard tetramethylsilane) as an external standard.

## 3 Results and Discussion

### 3.1 KNH_2_–RbH System

**Figure 2:**
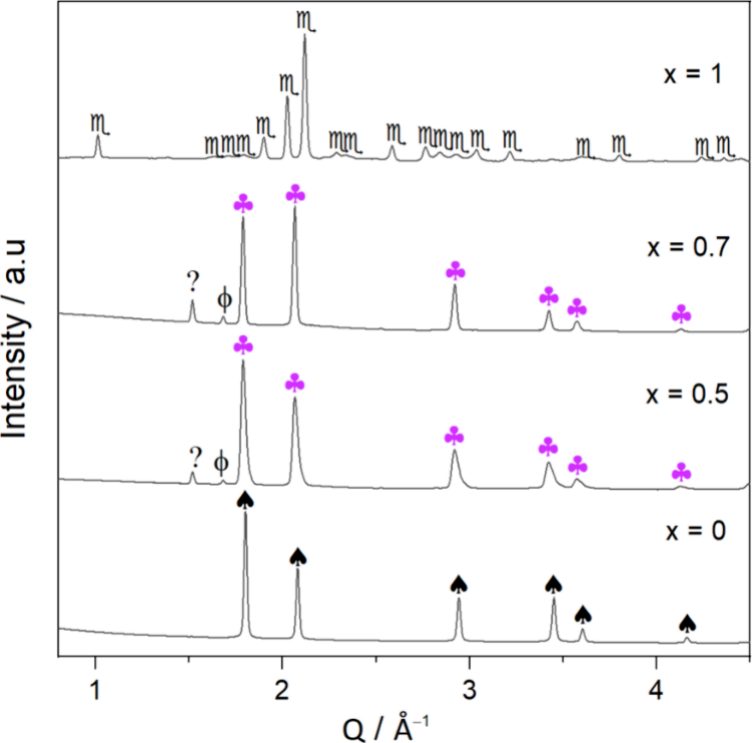
RT XRD of the annealed *x*KNH_2_ + (1 – *x*)RbH samples, with *x* = {0, 0.5, 0.7, 1}. 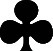 = Rb–K–N–H solid solution (*Fm*3̅*m*), 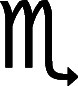 = KNH_2_ (*P*2_1_/*m*), 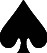 = RbH (*Fm*3̅*m*), ϕ = K metallic (*Im*3̅*m*), and ? = unknown phase.

Figure 2 shows the room temperature XRD patterns of the annealed *x*KNH_2_ + (1 – *x*)RbH samples, where *x* = {0, 0.5, 0.7, 1}. Note that in the XRD pattern of the compositions featuring *x* = {0.5, 0.7}, the reflections of KNH_2_ (monoclinic, s.g. *P*2_1_/*m*, labeled 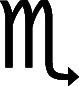) are not visible. This indicates that KNH_2_ reacted with RbH, accompanied by the formation of a single cubic phase (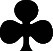, *s.g.Fm*3̅*m*). In addition, the metallic K (labeled ϕ) and an unknown phase (labeled ?) are also present. This may indicate a partial decomposition of reactants under given conditions. In addition, for the *x* = 0.5 composition, the peaks appear broadened, particularly at the high *Q*-values, suggesting an overlap of multiple phases due to incomplete reactions between the amide and hydride. To clarify these, in situ SR-PXD measurements were carried out.

**Figure 3:**
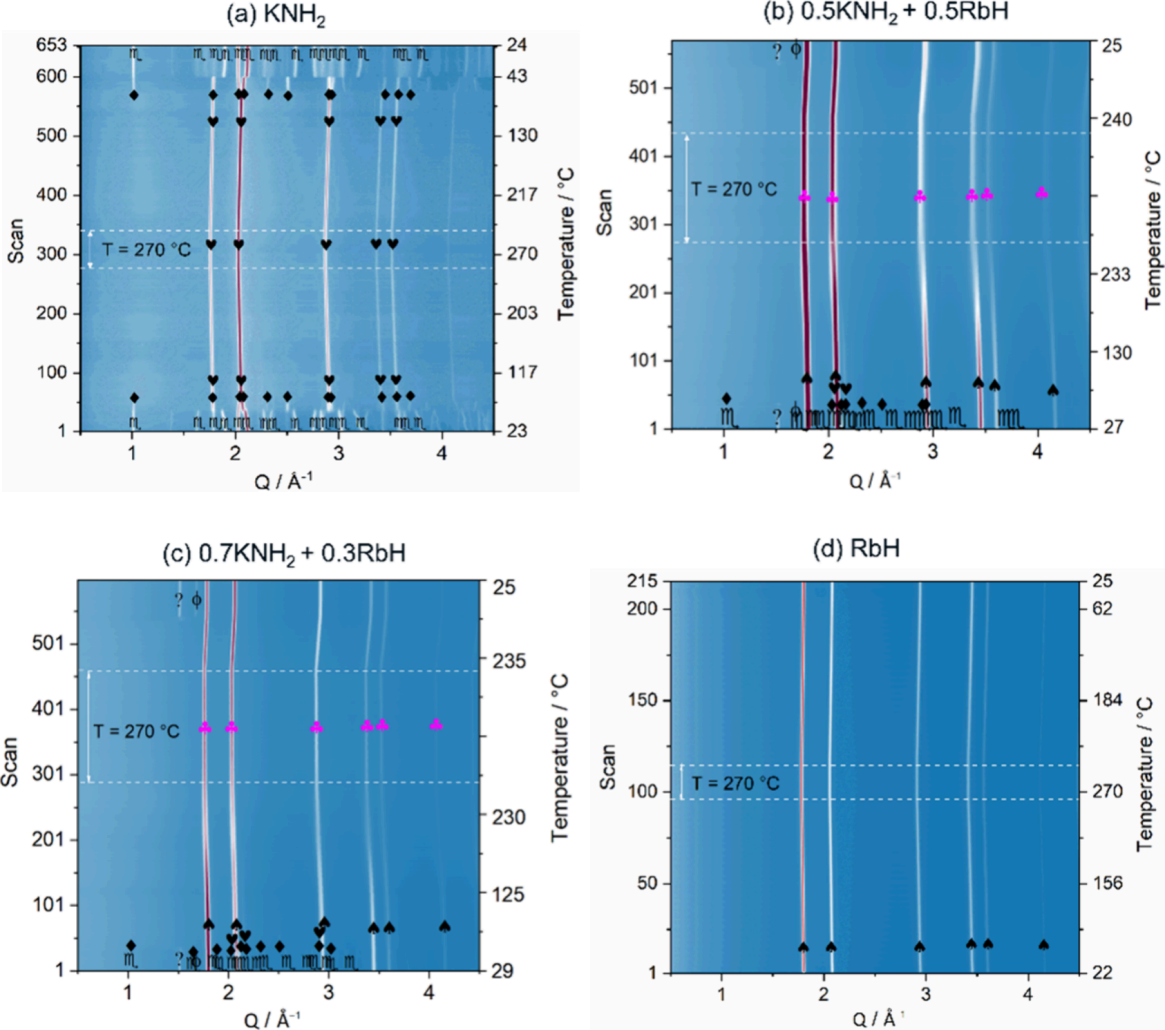
In situ SR-PXD data of the (a) KNH_2_, (b) 0.5KNH_2_ + 0.5RbH, (c) 0.7KNH_2_ + 0.3RbH, and (d) RbH. 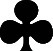 = Rb–K–N–H solid solution (*Fm*3̅*m*), 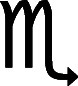 = KNH_2_ (*P*2_1_/*m*), ⧫ = KNH_2_ (*P*4/*nmm*), 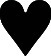 = KNH_2_ (*Fm*3̅*m*), 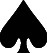 = RbH (*Fm*3̅*m*), ϕ = K (*Im*3̅*m*), and ? = unknown phase.

In order to assess the formation of the single cubic phase seen in Figure 2, as well as the microstructural evolution of the investigated material under temperature and pressure variations, in situ SR-PXD experiments were carried out on the initial and mixed samples. Figure 3 shows the in situ SR-PXD data of the *x*KNH_2_ + (1 – *x*)RbH samples, where *x* = {0, 0.5, 0.7, 1}. As can be seen in Figure 3, the in situ SR-PXD data of the KNH_2_ starting material show two phase transitions: the first one corresponds to the phase transformation of KNH_2_ from a monoclinic (s.g. *P*2_1_/*m*) to a tetragonal structure (s.g. *P*4/*nmm*, ⧫) at about 55 °C and the second one is the phase conversion of KNH_2_ from the tetragonal (s.g. *P*4/*nmm*) to the cubic (s.g. *Fm*3̅*m*, 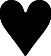) structure at about 75 °C, while RbH (s.g. *Fm*3̅*m*, 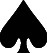) does not undergo structural changes during the heating and cooling periods. These observations agree with those reported in references and literatures.^16,31^ For mixed samples with *x* = {0.5, 0.7}, their in situ SR-PXD data appear similar, and both differ from the in situ SR-PXD data of the starting samples (KNH_2_ and RbH). At the beginning of the heating period, both *x* = 0.5 and *x* = 0.7 mixtures show the existence of monoclinic KNH_2_ (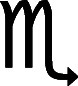), cubic RbH (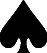). Besides, tiny peaks of metallic K (ϕ) and unknown phase (?) are also detected. Trying to understand the reason for the presence of metallic K and the unknown phase found for the mixed samples, the gas evolution for the *x* = 0.7 composition during the mixing under inert atmosphere of KNH_2_ and RbH was monitored by mass spectrometer (MS). As demonstrated in ESI-Figure S1, the investigation’s findings indicated signs of hydrogen and ammonia release during mixing. The presence of metallic K and an unknown phase, along with the gas evolution found by MS, indicates a partial decomposition of reactants. However, according to the Rietveld refinement, the cell parameter of the RbH-like structure for the *x* = 0.7 composition (ESI-Figure S2), *a*, is 6.0405617 Å, thus larger than the RbH cell parameter (*a* = 6.0363336 Å) of the pure sample (ESI-Figure S3). This increase corresponds to a volume expansion of RbH of 0.21%. This supports the assumption that the solid solution formation by the cation and anion substitution, which underlies the interaction between KNH_2_ and RbH, already occurred during grinding. As the temperature increases, phase transitions are found for KNH_2_ from the monoclinic (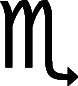) to the tetragonal (⧫) and then to the cubic (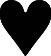) structure, as predicted, while the RbH peaks (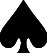) remain unchanged, like what was observed for the pure KNH_2_ and RbH samples. At *T* ∼ 160 °C, solid solution formation begins, corresponding to the disappearance of the cubic KNH_2_ diffraction peaks and the shift of the cubic RbH peaks toward the lower *Q* values, indicating a gradual transformation from the RbH to the solid solution structure. This process continues during the heating period and is completed at 270 °C, with the observation of a complete single cubic solid solution phase (s.g. *Fm*3̅*m*, 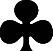). This phase (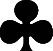) is stable on cooling and at RT after the measurement. No Bragg peaks of initial materials (i.e., monoclinic KNH_2_ and cubic RbH) are detected, suggesting the stability of the solid solution. In addition, small peaks of elemental K (ϕ) and unknown phase (?) are back at RT after the measurement for both *x* = 0.5 and *x* = 0.7 compositions, like what was observed in the ex situ XRD (see Figure 2).

### 3.2 RbNH_2_–KH System

**Figure 4:**
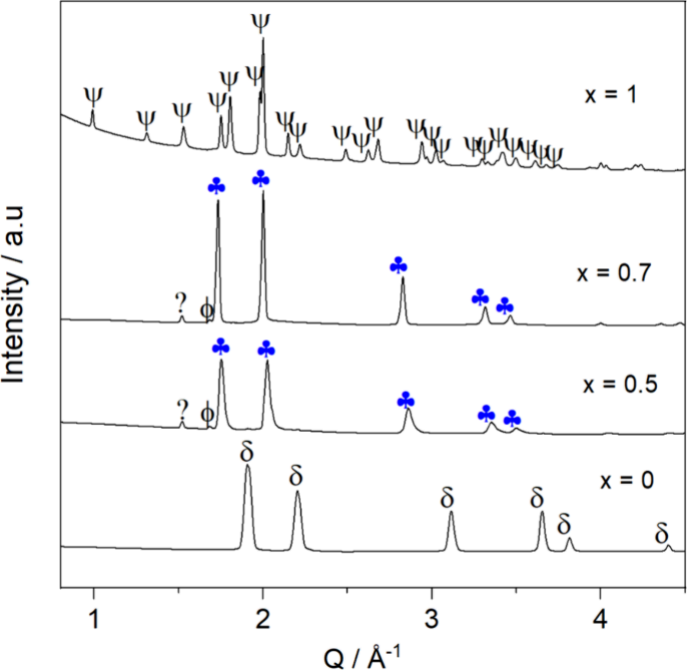
RT XRD data of the *x*RbNH_2_ + (1 – *x*)KH samples after annealing at 270 °C, with *x* = {0, 0.5, 0.7, 1}. ψ = RbNH_2_ (*P*2_1_/*m*), δ = KH (*Fm*3̅*m*), 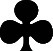 = Rb–K–N–H solid solution (*Fm*3̅*m*), ϕ = K (*Im*3̅*m*), and ? = unknown phase.

Similarly, the room temperature XRD diffractograms of the *x*RbNH_2_ + (1 – *x*)KH samples after being annealing at 270 °C are shown in Figure 4. The data show that for the compositions *x* = {0.5, 0.7}, the Bragg peaks of the RbNH_2_ (ψ) and KH (δ) phases are not detected, but instead, a single cubic phase (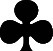, s.g. *Fm*3̅*m*) is observed, indicating the dissolution of RbNH_2_ and KH within the structure. Small peaks related to elemental K (ϕ) and an unknown phase (?) are also observed, similarly to the KNH_2_–RbH system (Section 3.1). It should be noted that peak broadening is also observed for the *x* = 0.5 composition. This will be discussed in detail in the Rietveld refinements of XRD data, which are displayed in Figures 6 and 7.

**Figure 5:**
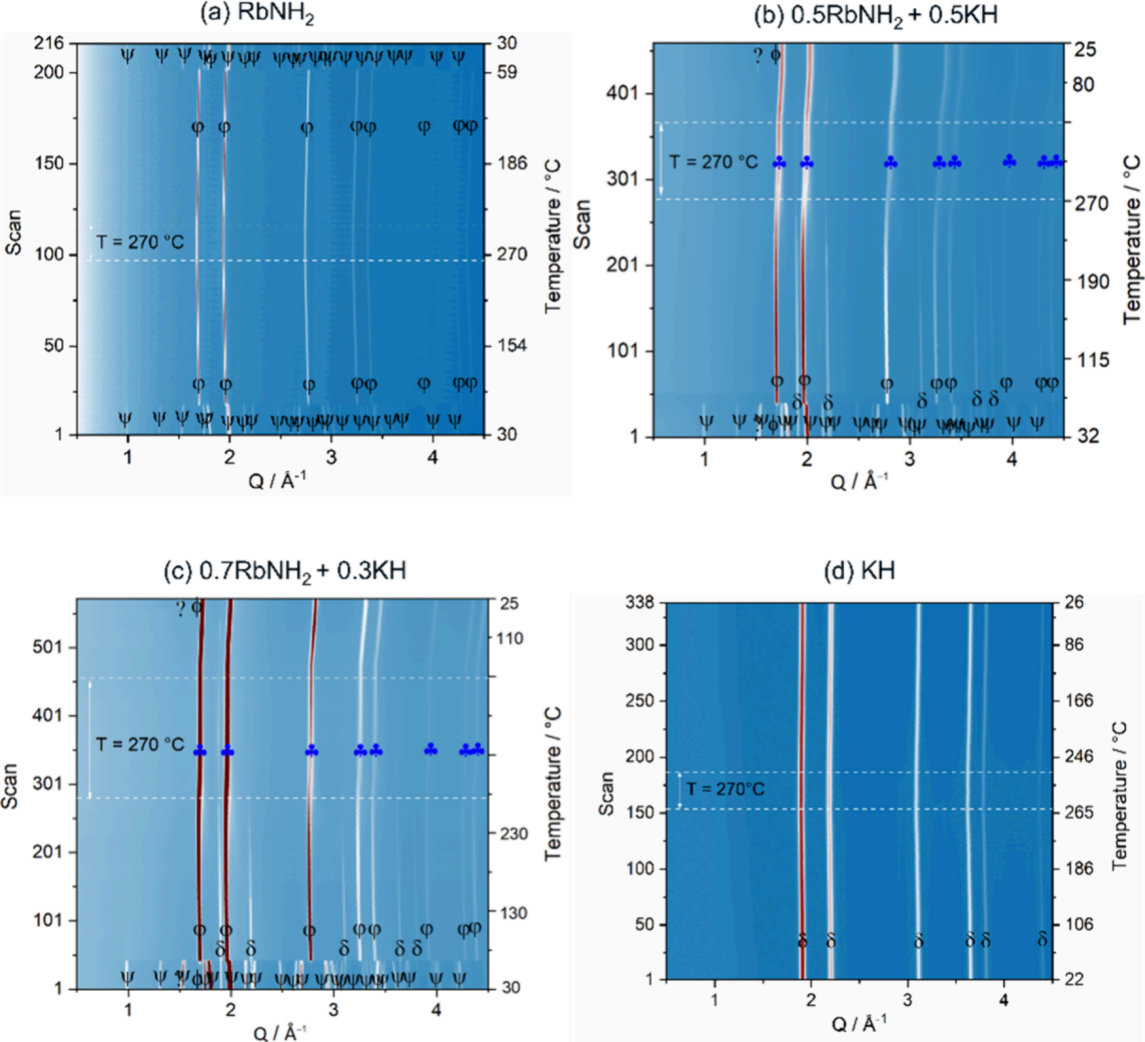
In situ SR-PXD data of the (a) RbNH_2_, (b) 0.5RbNH_2_ + 0.5KH, (c) 0.7RbNH_2_ + 0.3KH, and (d) KH. ψ = RbNH_2_ (*P*2_1_/*m*), φ = RbNH_2_ (*Fm*3̅*m*), δ = KH (*Fm*3̅*m*), 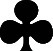 = Rb–K–N–H solid solution (*Fm*3̅*m*), ϕ = K (*Im*3̅*m*), and ? = unknown phase.

As before, the phase evolution under real conditions was monitored with the conduction of in situ SR-PXD measurements. As shown in Figure 5, the in situ SR-PXD data of the *x*RbNH_2_ + (1 – *x*)KH samples, where *x* = {0, 0.5, 0.7, 1}, were also collected. The in situ SR-PXD data of starting material RbNH_2_ show a phase transition of RbNH_2_ from a monoclinic (s.g. *P*2_1_/*m*, labeled ψ) to a cubic structure (s.g. *Fm*3̅*m*, labeled φ) at around 68 °C, while the RT-cubic structure of KH (s.g. *Fm*3̅*m*, labeled δ) does not undergo a phase transition under the given experimental conditions, similar to the RbH. These results agree with those reported in refs (16 and 31). For compositions with *x* = {0.5, 0.7}, their in situ SR-PXD data show similarities, i.e., both show the transformation of RbNH_2_ from the monoclinic (ψ) to the cubic (φ) structure at about 65 °C during heating, as expected. Subsequently, the mutual dissolution of RbNH_2_ and KH takes place, accompanied by the substitution process of cubic RbNH_2_ (φ) and cubic KH (δ) peaks observed starting from a temperature of about 150 °C and further increased during the isothermal period. As a result, the completion of solubility is achieved and the formation of a single cubic solid solution is observed for both compositions with *x* = {0.5, 0.7}, similar to the KNH_2_–RbH system. In addition, metallic K (ϕ) and an unknown phase (?) are observed at room temperature, similarly to the KNH_2_–RbH system.

**Figure 6:**
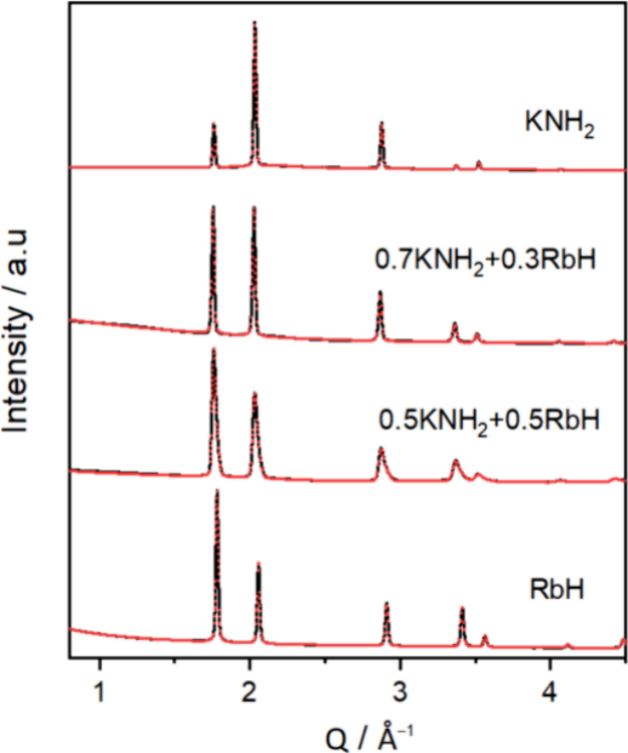
Rietveld refinements of the in situ SR-PXD data collected at 270 °C for *x*KNH_2_ + (1 – *x*)RbH samples, where *x* = {0, 0.5, 0.7, 1}.

**Figure 7:**
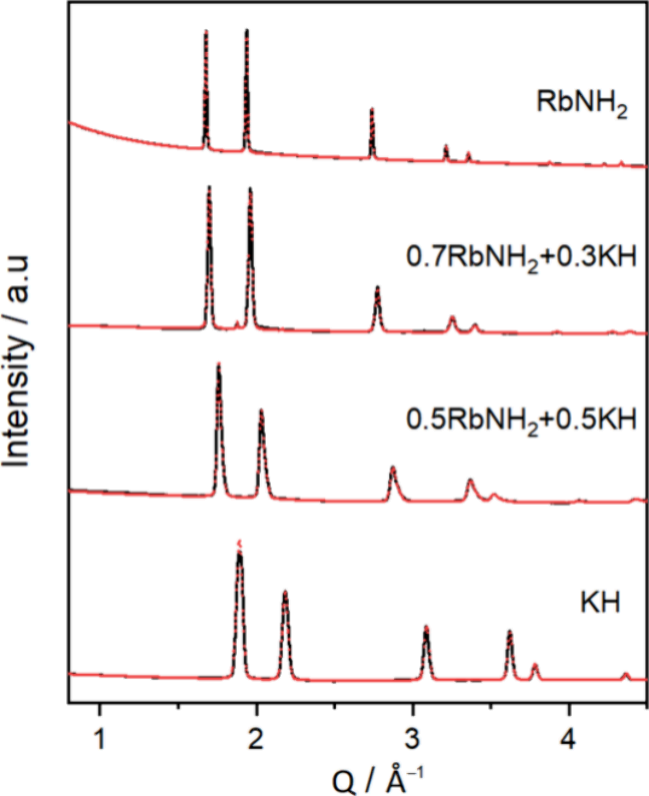
Rietveld refinements of the in situ SR-PXD data collected at 270 °C for *x*RbNH_2_ + (1 – *x*)KH samples, where *x* = {0, 0.5, 0.7, 1}.

To elucidate phase compositions in the mixed samples, Rietveld refinement of in situ SR-PXD data was performed (Rietveld refinement details can be found in ESI-Figure S4). Figure 6 and Table 1 present the Rietveld refinements of diffraction data collected at 270 °C and cell parameters of phases within the KNH_2_–RbH system. For the *x* = {0.5, 0.7} compositions, the results confirmed the formation of a single cubic solid solution this these mixtures, also considering the partial desorption of ammonia and hydrogen during heating. Additionally, unreacted RbH is also observed for the *x* = 0.5 sample, indicating a not complete solubility of amide and hydride at this temperature. Similarly, for the RbNH_2_–KH system, Rietveld refinement of XRD data is performed, as depicted in Figure 7 and Table 2. For the *x* = 0.7 sample, a single cubic solid solution of amide and hydride is formed, while for the *x* = 0.5 sample, an overlap of two solid solution phases corresponding to broader peaks observed in the XRD diffraction data is detected. Additionally, unreacted KH is also observed. Tables 1 and 2 present the phase compositions and lattice parameters of phases in both systems. The existence of cubic polymorphs of KNH_2_ and RbNH_2_ at temperature higher than 75 °C and their structural similarities with RbH and KH (e.g., cation and anion radii: *r*_K+_ = 1.52 Å, *r*_Rb+_ = 1.66 Å, *r*_NH2-_= 1.73 Å, *r*_H–_ = 1.53 Å and cell parameters at 270 °C: KNH_2_ (*a* = 6.19766 Å), RbNH_2_ (*a* = 6.10687 Å), KH (*a* = 5.74913 Å), and RbH (*a* = 6.10687 Å)) pave the path to the formation of mixed solid solutions. It is observed that for the KNH_2_–RbH system, the cubic KNH_2_ likely dissolves into the cubic RbH structure, as seen by the disappearance of the KNH_2_ diffraction peaks and the change in the RbH peaks (in situ XRD data in Figure 3). In contrast, the cubic KH dissolves into the cubic RbNH_2_, detected by the shift of RbNH_2_ peaks at *T* ∼ 270 °C, when the solubility takes place (in situ XRD data in Figure 5). Besides, the diffraction data of the two systems (i.e., KNH_2_–RbH (Figure 6) and RbNH_2_–KH (Figure 7)) also show that the diffraction peaks are shifted, and their relative intensity varies gradually with increasing the amount of amide (i.e., *x*). This is indicative of the expected ionic substitution and change of the unit cell (see Tables 1 and 2).

**Table 1:** Lattice Parameters of the Solid Solution in the KNH_2_–RbH System Obtained from the Rietveld Refinement of the SR-PXD Patterns Acquired at 270 °C

system 1	phase compositions	unit cell (*a*/Å)
KNH_2_	KNH_2_	6.19766
0.7KNH_2_ + 0.3RbH	Rb_0.8_K_0.2_(NH_2_)_0.4_ H_0.6_ (100 wt %)	6.17582
0.5KNH_2_ + 0.5RbH	Rb_0.9_K_0.1_(NH_2_)_0.44_H_0.56_ (78.9 wt %)	6.17899
	RbH-like cubic (21.1 wt %)	6.10120
RbH	RbH	6.10687

**Table 2:** Lattice Parameters of the Solid Solution in the RbNH_2_–KH System Obtained from the Rietveld Refinement of the SR-PXD Patterns Acquired at 270 °C

system 2	phase compositions	unit cell (*a*/Å)
RbNH_2_	RbNH_2_	6.48491
0.7RbNH_2_ + 0.3KH	Rb_0.77_K_0.23_(NH_2_)_0.68_H_0.32_ (97.4 wt %)	6.38822
	KH-like cubic (2.6 wt %)	5.78233
0.5RbNH_2_ + 0.5KH	Rb_0.94_K_0.06_(NH_2_)_0.91_H_0.09_ (48.2 wt %)	6.32481
	K_0.99_(Rb_0.01_)NH_2_ (48 wt %)	6.23548
	KH-like cubic (3.8 wt %)	5.77403
KH	KH	5.74913

The vibrational frequencies of the NH_2_ group in the pure amides and mixed amide-hydride samples were characterized by FT-IR. As shown in ESI-Figure S5a for the KNH_2_–RbH system, the stretching vibrational modes of the N–H bonds in KNH_2_ are observed at 3253 and 3207 cm^–1^. These signals slightly shift to lower frequencies in the mixed amide-hydride samples, probably due to volume expansion, which increases the N–H bond length, and the ionic substitution (K with Rb). In addition, the magnitude of these bands is weaker in the mixed samples with a reduced amount of amide compared to the original KNH_2_, indicating the decrease of the dipole moment caused by the reduction of the negative charge on the nitrogen atom induced by a substituent such as hydrogen. This observation is similar to what has been observed in the Cs–N–H system^32^ and for C–H bonds.^37,38^ Likewise, a similar observation on the vibrational modes is also found for the RbNH_2_–KH system, where the intensity of the signals of the stretching modes decreases because of the low amount of amide in the samples (ESI-Figure S5b).

**Figure 8:**
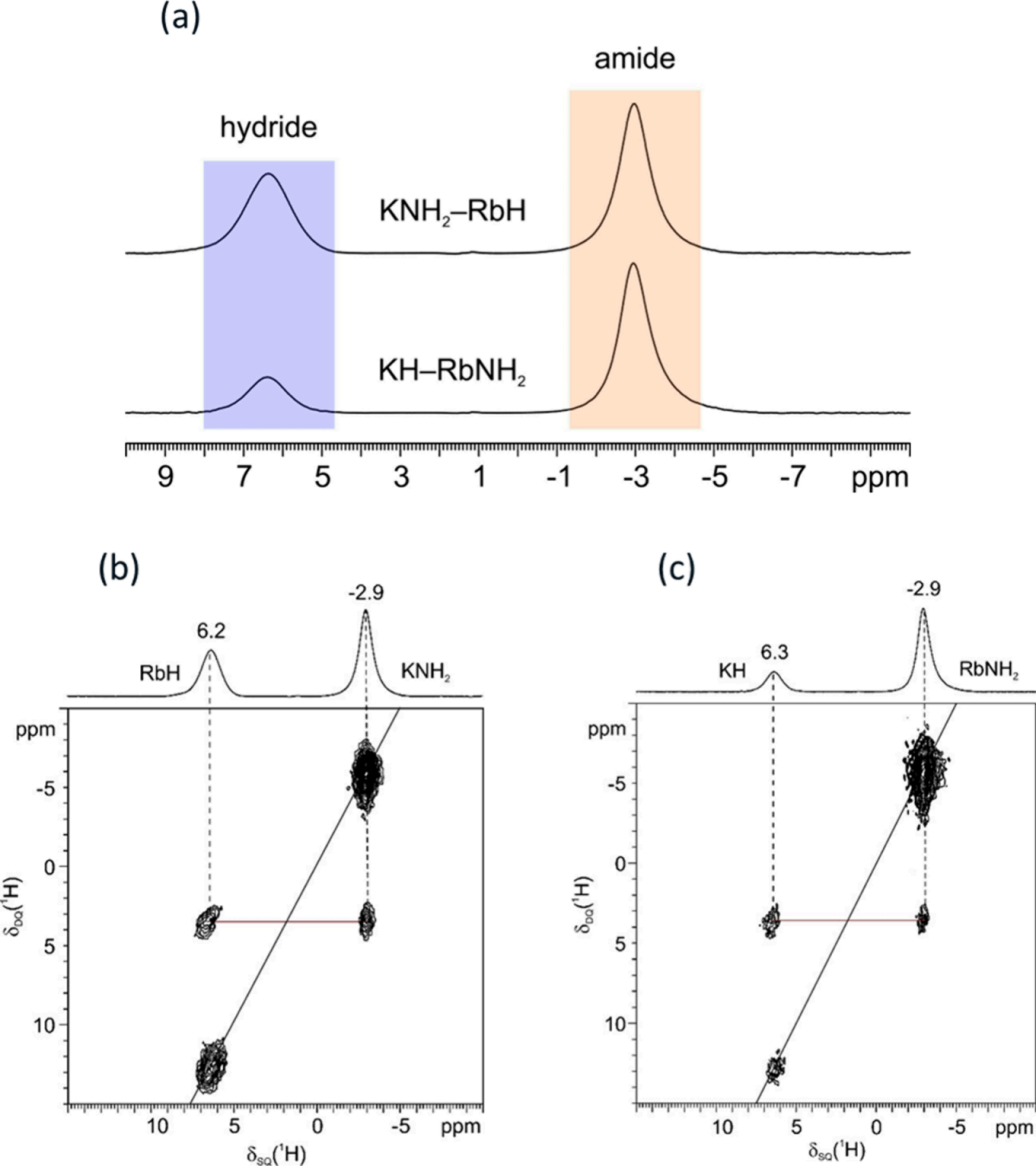
(a) ^1^H (600.13 MHz) MAS SSNMR spectra of the 0.5KNH_2_ + 0.5RbH and 0.5RbNH_2_ + 0.5KH compositions, acquired at probe temperature at a spinning speed of 20 kHz; 2D ^1^H (600.13 MHz) DQ MAS SSNMR spectra of the (b) 0.5KNH_2_ + 0.5RbH and (c) 0.5RbNH_2_ + 0.5KH systems, recorded at a spinning speed of 20 kHz at probe temperature. Red lines highlight the DQ correlation between the amide and hydride signals in both systems.

It is noteworthy that although partial desorption of ammonia and hydrogen is observed, the formation of mixed metal amide-hydride solid solution is evident. Further confirmation of the solid solution formation for the KNH_2_–RbH and RbNH_2_–KH systems was achieved by solid-state NMR measurements. Figure 8a shows the ^1^H MAS spectra of the 0.5KNH_2_ + 0.5RbH and 0.5RbNH_2_ + 0.5KH systems. In both spectra, similar chemical shifts for the hydride (6.2–6.3 ppm) and amide (−2.9 ppm) anions are observed, indicating the similarity of the chemical environment between these two mixed samples. Important note that, the presence of imide is not notied, thi

A direct assessment of the solid solution formation was provided by the 2D ^1^H DQ MAS spectra, as shown in Figure 8b,c. For the 0.5KNH_2_ + 0.5RbH sample (Figure 8b), the DQ correlation between the amide (δ_SQ_ = −2.9 ppm) and hydride (δ_SQ_ = 6.2 ppm) signals is observed at δ_DQ_ = 3.3 ppm. This implies that the protons of KNH_2_ and RbH are in close spatial proximity to each other, i.e., within 3.5 Å, which implies that they belong to a homogeneous phase.^39,40^ A similar correlation is also observed for the 0.5KNH_2_ + 0.5RbH sample, where a DQ correlation between the amide (δ_SQ_ = −2.9 ppm) and the hydride (δ_SQ_ = 6.3 ppm) signals is found at δ_DQ_ = 3.4 ppm (Figure 8c), also indicating the intimate spatial proximity of the RbNH_2_ and KH protons. The results of the in situ/ex situ XRD and SSNMR measurements provide adequate evidence to confirm the formation of solid solutions for both the KNH_2_–RbH and RbNH_2_–KH systems.

### 3.3 RbNH_2_–CsH and CsNH_2_–KH Systems

**Figure 9:**
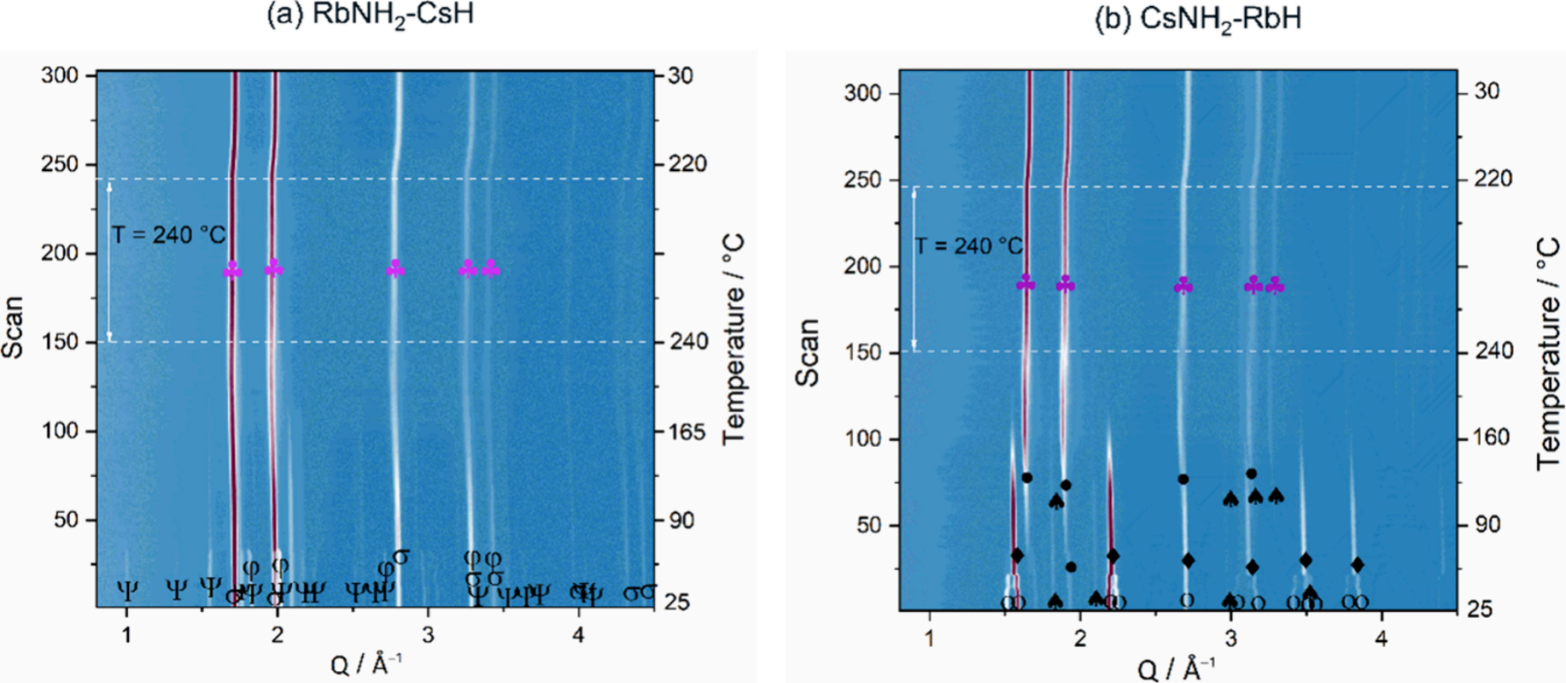
In situ SR-PXD data of the RbNH_2_–CsH (a) and CsNH_2_–RbH (b) composites. Ψ = RbNH_2_ (*P*2_1_/*m*), φ = RbNH_2_ (*Fm*3̅*m*), σ = CsH (*Fm*3̅*m*), 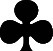 = RbNH_2_–CsH solid solution (*Fm*3̅*m*). ο = CsNH_2_ (*P*4/*nmm*), ⧫ = CsNH_2_ (*Pm*3̅*m*), • = CsNH_2_ (*Fm*3̅*m*), 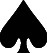 = RbH (*Fm*3̅*m*), and 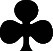 = CsNH_2_–RbH solid solution (*Fm*3̅*m*).

Extending the investigation in this line of research, we observed that solid solutions were also formed in the RbNH_2_–CsH and CsNH_2_–RbH systems. Figure 9 shows the phase evolution in these mixed amide-hydride composites in the temperature range from RT to 240 °C. As shown in Figure 9a for the in situ SR-PXD data of the RbNH_2_–CsH composite, a phase transformation of RbNH_2_ occurs, from a monoclinic (s.g. *P*2_1_/*m*) to a cubic structure (s.g. *Fm*3̅*m*), while the cubic CsH structure (s.g. *Fm*3̅*m*) does not undergo any phase transition. These observations are similar to those reported earlier in this work and in ref (32). It is noted that at *T* > 65 °C, both RbNH_2_ and CsH have similar crystal structures (s.g. *Fm*3̅*m*), which might facilitate the ion mobility and consequently the formation of the solid solution. At the temperature of 240 °C, the disappearance of the cubic RbNH_2_ phase and the change of the cubic CsH peaks are observed, indicating the mutual solubility of the RbNH_2_ and CsH to form a solid solution with the same space group. This single cubic solid solution is stable and remains unchanged during cooling. Similarly, for the CsNH_2_–RbH composite, the in situ SR-PXD data seen in Figure 9b show the phase transition of CsNH_2_ from a tetragonal structure (s.g. *P*4/*nmm*) to cubic ones (s.g. *Pm*3̅*m* and *Fm*3̅*m*), comparable to what was reported in ref (32), while the cubic RbH structure (s.g. *Fm*3̅*m*) remains stable. A single cubic phase (s.g. *Fm*3̅*m*) is observed at the isothermal temperature of 240 °C, indicating the mutual solubility of RbH and CsNH_2_. This CsNH_2_–RbH cubic solid solution is stable during cooling. The results derived from the in situ SR-PXD data of RbNH_2_–CsH and CsNH_2_–RbH are comparable to those obtained for the KNH_2_–RbH and RbNH_2_–KH systems. Therefore, it is reasonable to assume that the formation of a solid solution was similarly achieved for these systems.

## 4 Conclusions

In this work, the formation of solid solutions in the amide-hydride system of alkali metals (K, Rb, and Cs), namely, KNH_2_–RbH, RbNH_2_–KH, RbNH_2_–CsH, and CsNH_2_–RbH, has been elucidated by various techniques (i.e., ^1^H 1D and 2D SSNMR, FT-IR, and ex situ/in situ XRD), leading to the following conclusive points:

Due to the structural and physicochemical similarities between MNH_2_ amide and MH hydride, the mixed metal solid solutions are formed by both cation and anion exchange.
The formed MNH_2_-MH solid solutions such as RbNH_2_–KH, KNH_2_–RbH, RbNH_2_–CsH, etc., could replace ionic hydrides (KH, RbH, and CsH) as additives for hydrogen storage systems. Follow-up research will be conducted to investigate the hydrogen storage properties of the Mg(NH_2_)_2_-2LiH system in the presence of mixed alkali amide-hydride solid solutions.
It is worth noting that the formation of mixed metal amide-hydride solid solutions with an expanded lattice could lead to disorder of the crystal structure (due to the size difference between cations and anions substituted) and possibly facilitate the ion transport. This aspect will be further investigated in a future work.
The findings of this work, in conjunction with our earlier studies on the amide-hydride solid solutions, serve as the foundation for additional research aimed at expanding this area of study and identifying analogies with other systems.

## Supporting Information Available

MS data, Rietveld refinement data, and FT-IR spectra (PDF)
