# Supplementary material for: Mixed Metal Amide-Hydride Solid Solutions for Potential Energy Storage Applications
Source: Inorg Chem. 2024 May 30;63(24):11233–41. doi: 10.1021/acs.inorgchem.4c01016 (PMC11186013; doi:10.1021/acs.inorgchem.4c01016)
Supplement: Supplementary file 1 — ic4c01016_si_001.pdf [file ic4c01016_si_001.pdf]

Supporting Information

Mixed Metal Amide-Hydride Solid Solutions for Potential Energy Storage Applications

*Thi Thu Le[a, *], Simone Bordignon[b], Michele R. Chierotti[b], Yuanyuan Shang[a], Alexander Schökel[c], Thomas Klassen[a,d], Claudio Pistidda[a, *]*

[a]Institute of Hydrogen Technology, Helmholtz-Zentrum hereon GmbH, Max-Planck-Straße 1, D-21502 Geesthacht, Germany.

[b]Department of Chemistry - University of Torino, V. P. Giuria 7, I-10125, Torino, Italy.

[c]Deutsches Elektronen-Synchrotron DESY, Notkestraße 85, D-22607 Hamburg, Germany.

[d]Helmut Schmidt University, Holstenhofweg 85, D-22043 Hamburg, Germany.

*Corresponding authors: Thi Thu Le (thi.le@hereon.de); Claudio Pistidda (claudio.pistidda@hereon.de)

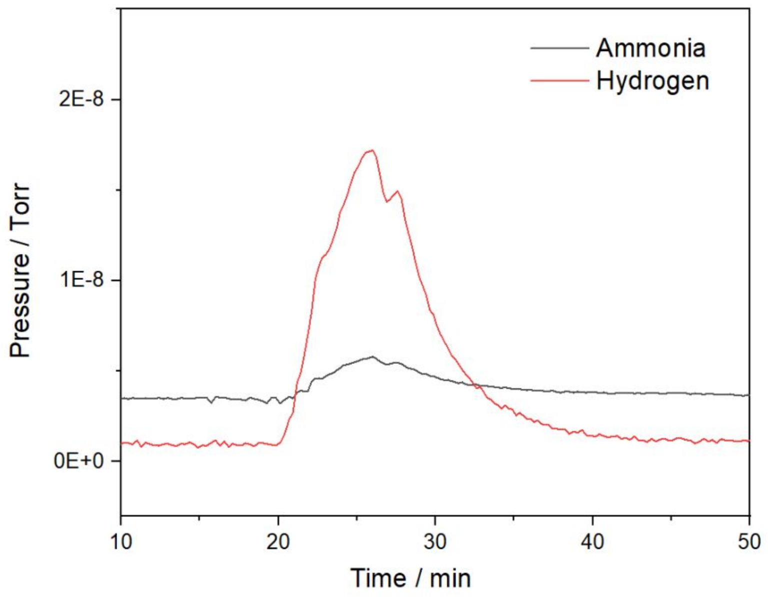

Figure S1. Gas evolution detected by mass spectrometer for the $0.7KNH_2+0.3RbH$ during mixing.

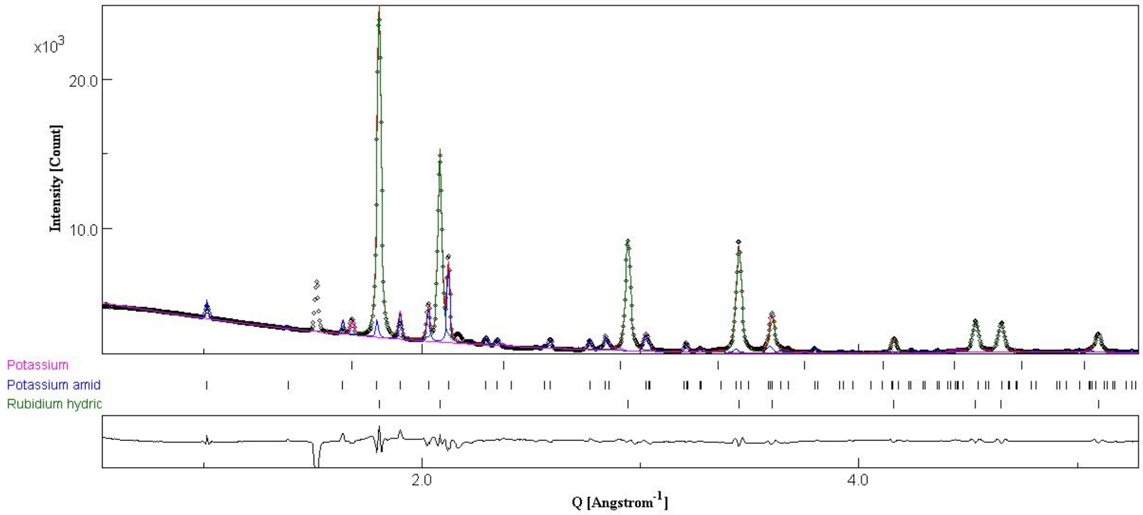

Figure S2. Rietveld refinement of the room temperature *in-situ* SP-PXD of $0.7KNH_2+0.3RbH$ sample. Cell parameter of the RbH-like phase, $a = 6.0405617$ Å. $R_{wp} = 5.8$ %.

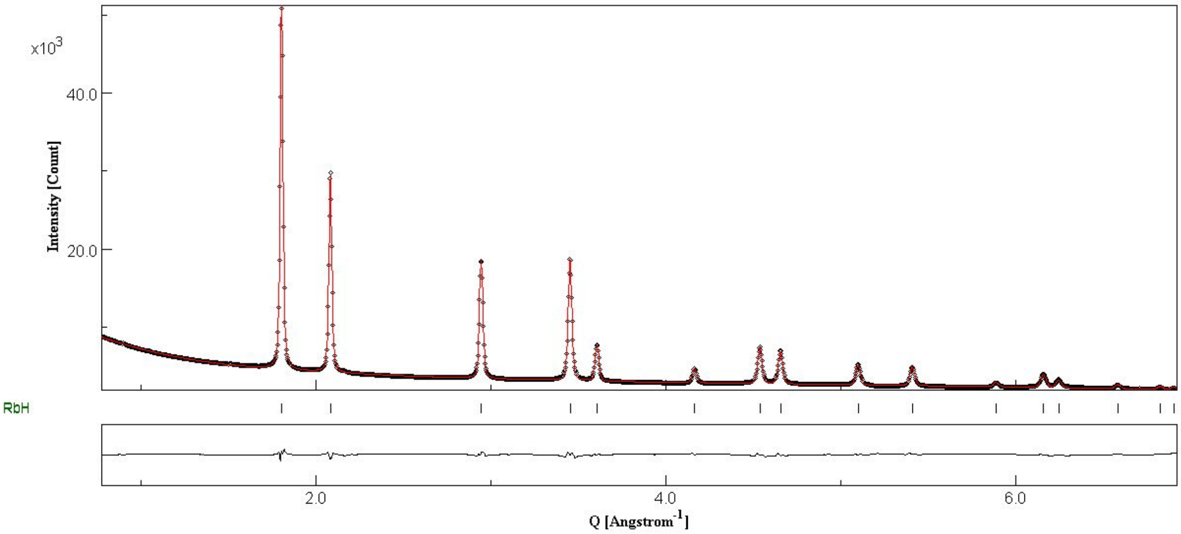

Figure S3. Rietveld refinement of the room temperature *in-situ* SP-PXD of the RbH sample. Cell parameter of the RbH: $a = 6.0363336$ Å. $R_{wp} = 2.1$ %.

(a) $RbNH_2$ (sigma = 0.80, $R_{wp} = 1.33$ %, $R_{exp} = 1.66$ %)

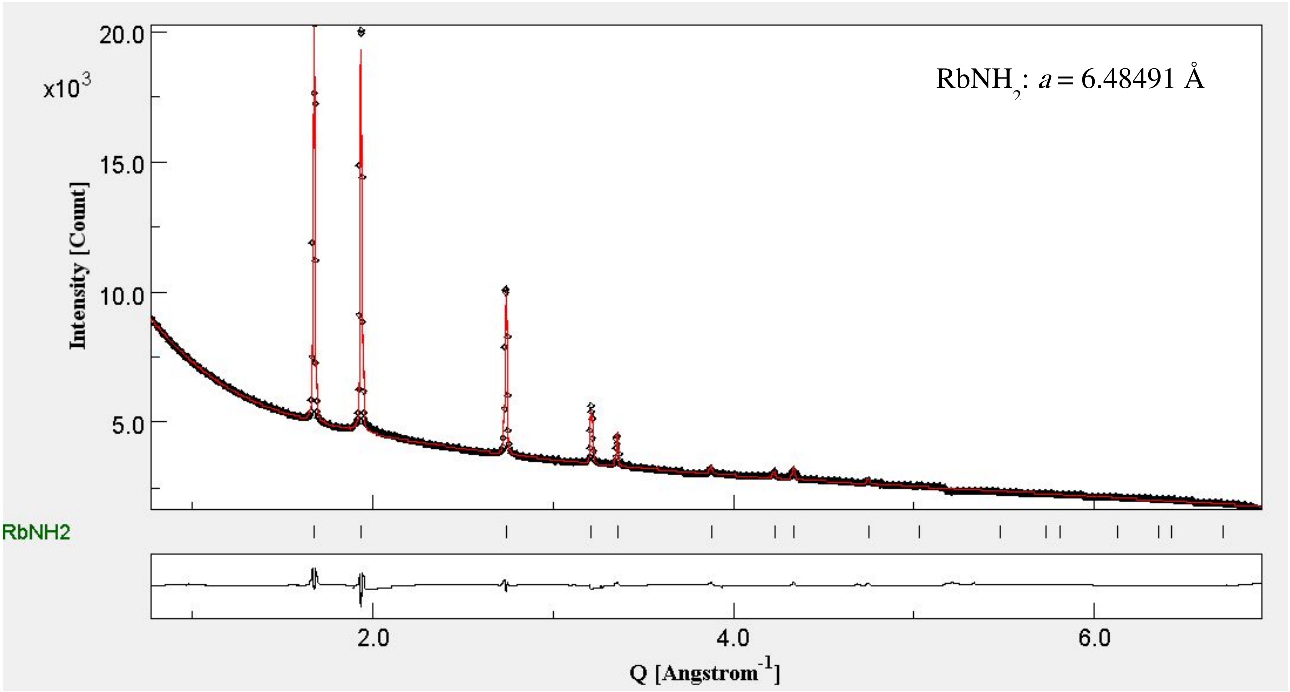

(b) KH (sigma = 2.18, R_{wp} = 4.14 %, R_{exp} = 1.90 %)

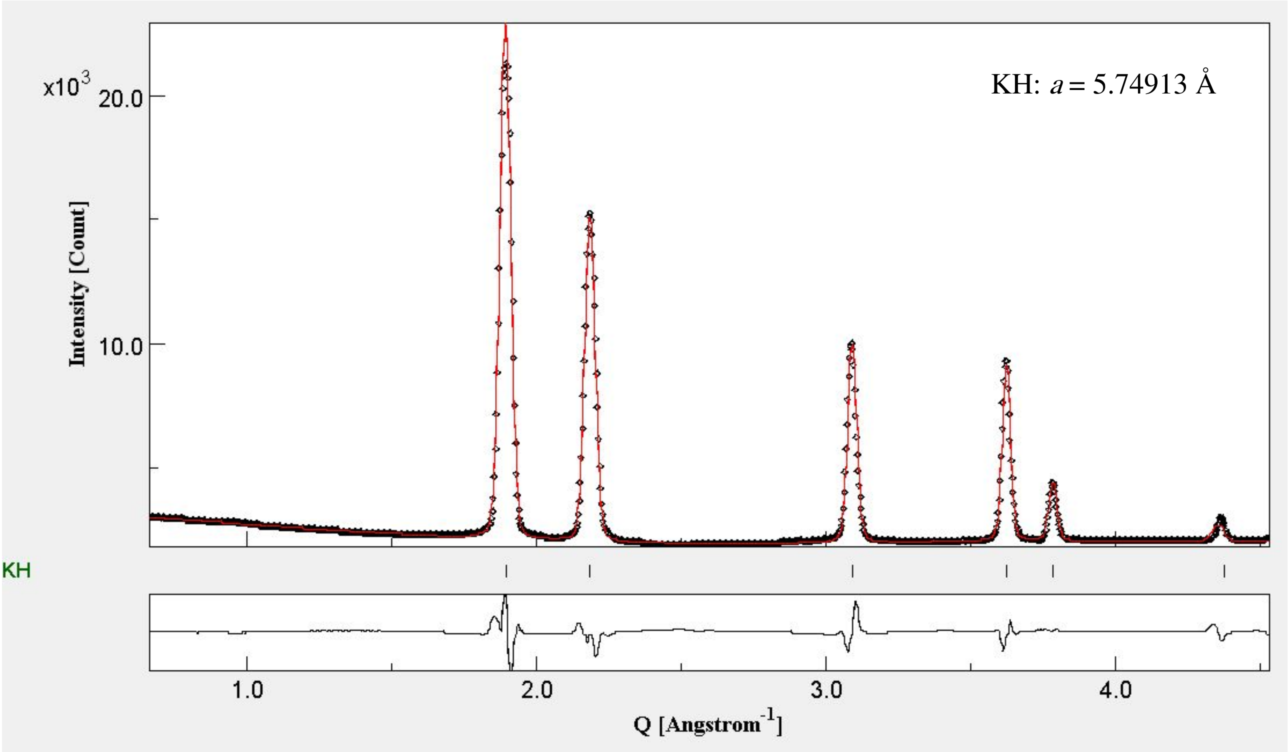

(c) 0.7$RbNH_2$+0.3KH (sigma = 2.0, R_{wp} = 3.24 %, R_{exp} = 1.62 %)

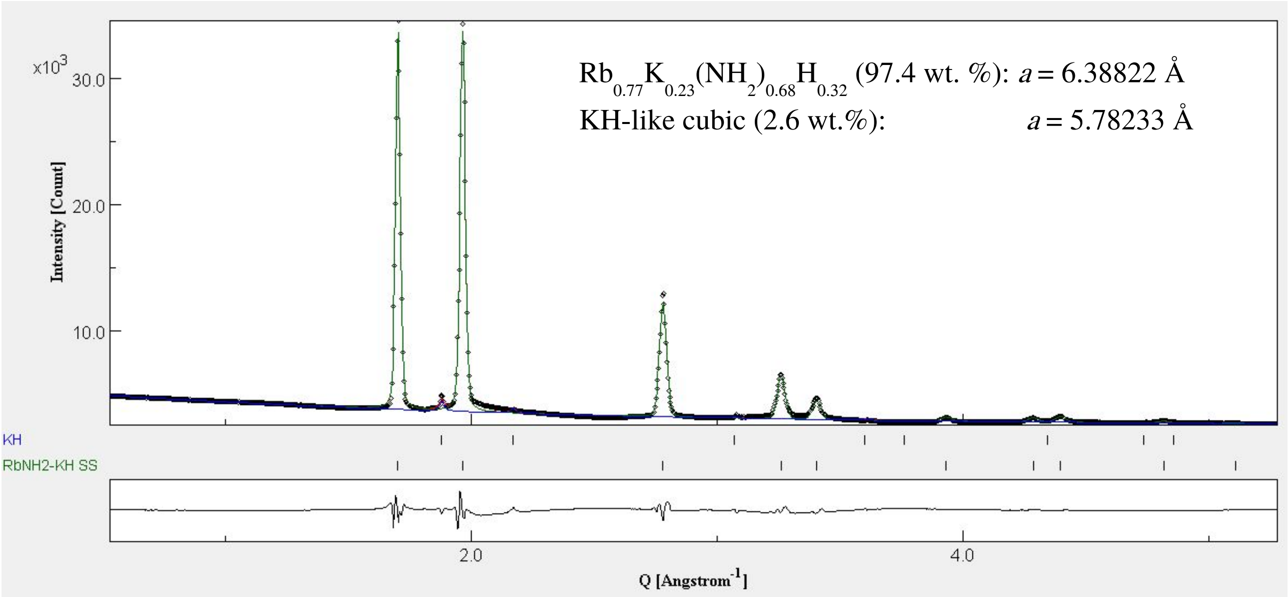

(d) $0.5RbNH_2$+0.5KH (sigma = 1.30, R_{wp} = 2.30 %, R_{exp} = 1.77 %)

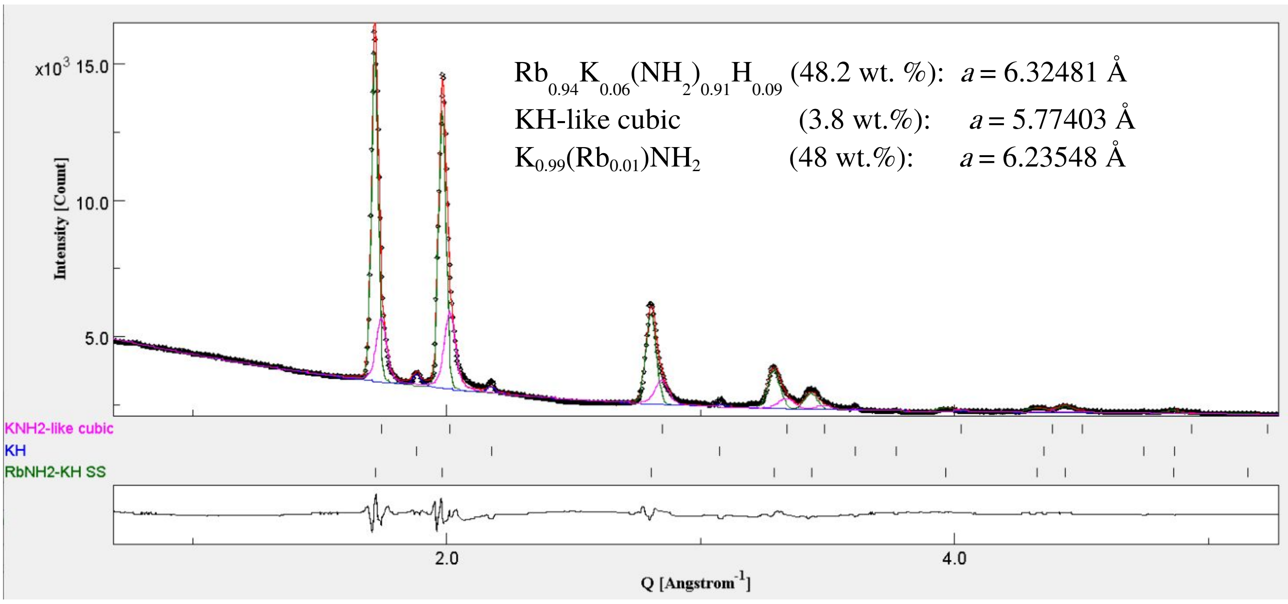

(e) KNH_2 (sigma = 0.51, R_{wp} = 3.58 %, R_{exp} = 7.07 %)

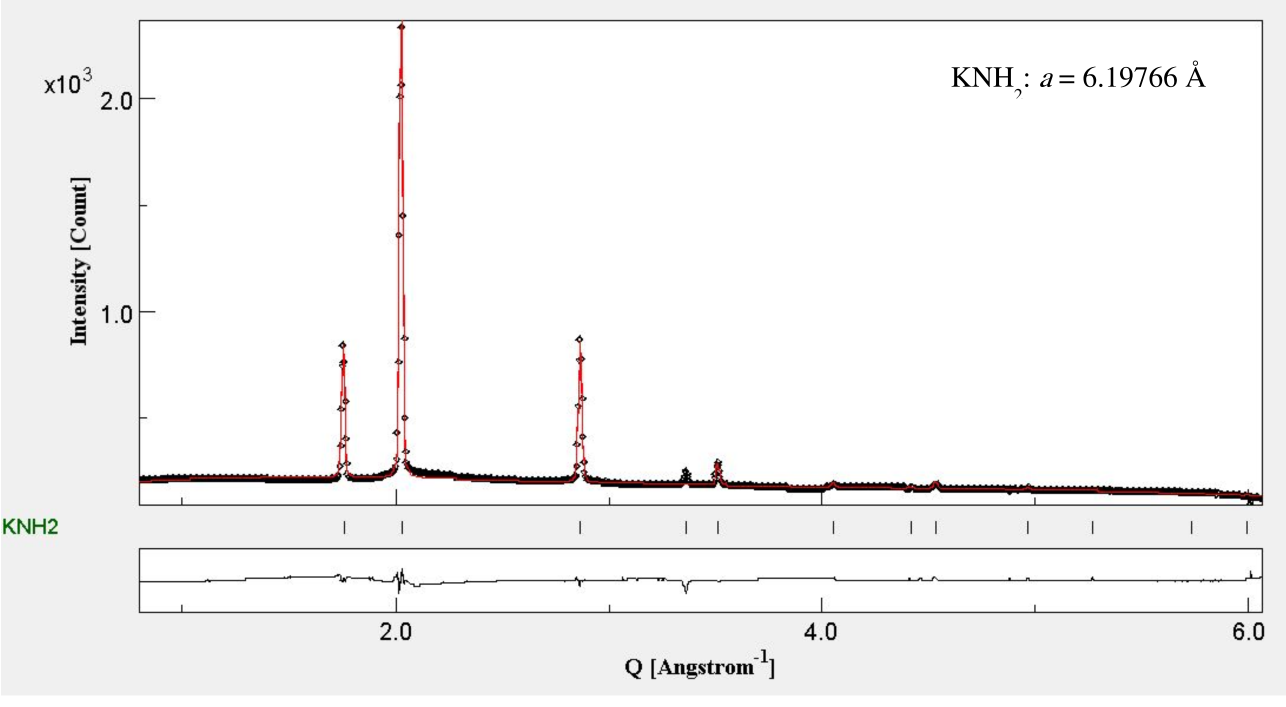

(f) RbH (sigma = 1.25, R_{wp} = 1.98 %, R_{exp} = 1.57 %)

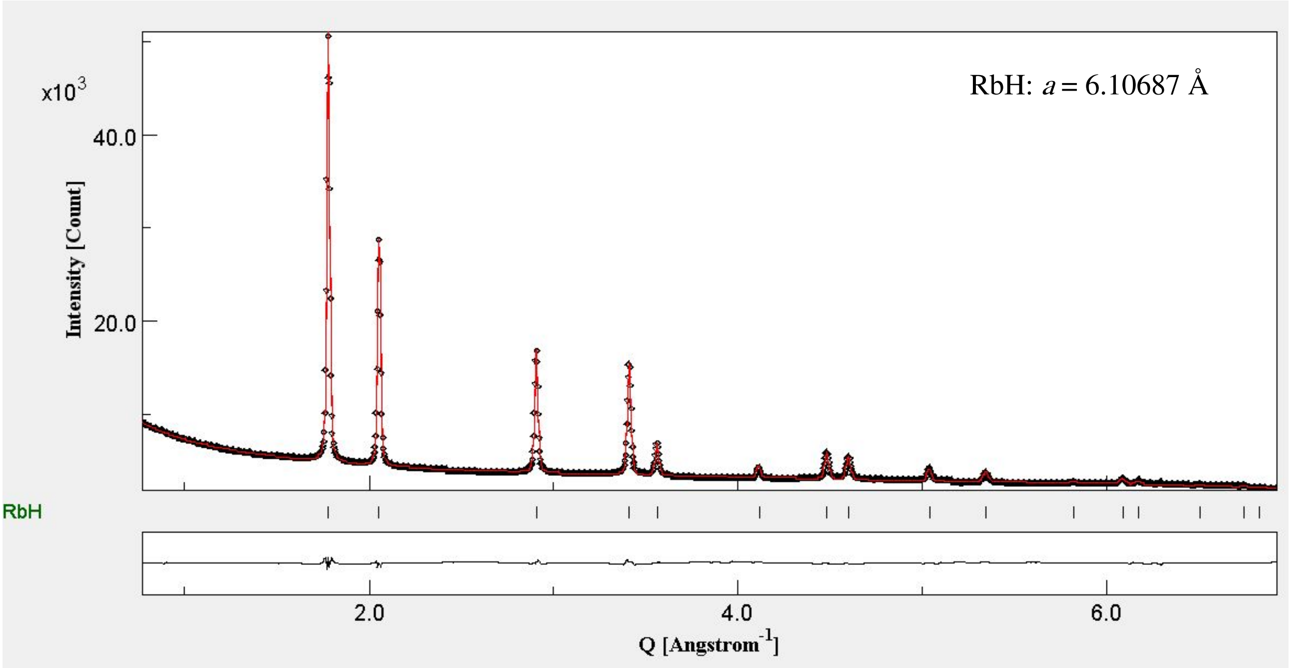

(g) 0.7KNH_2+0.3RbH (sigma = 1.41, R_{wp} = 2.62 %, R_{exp} = 1.84 %)

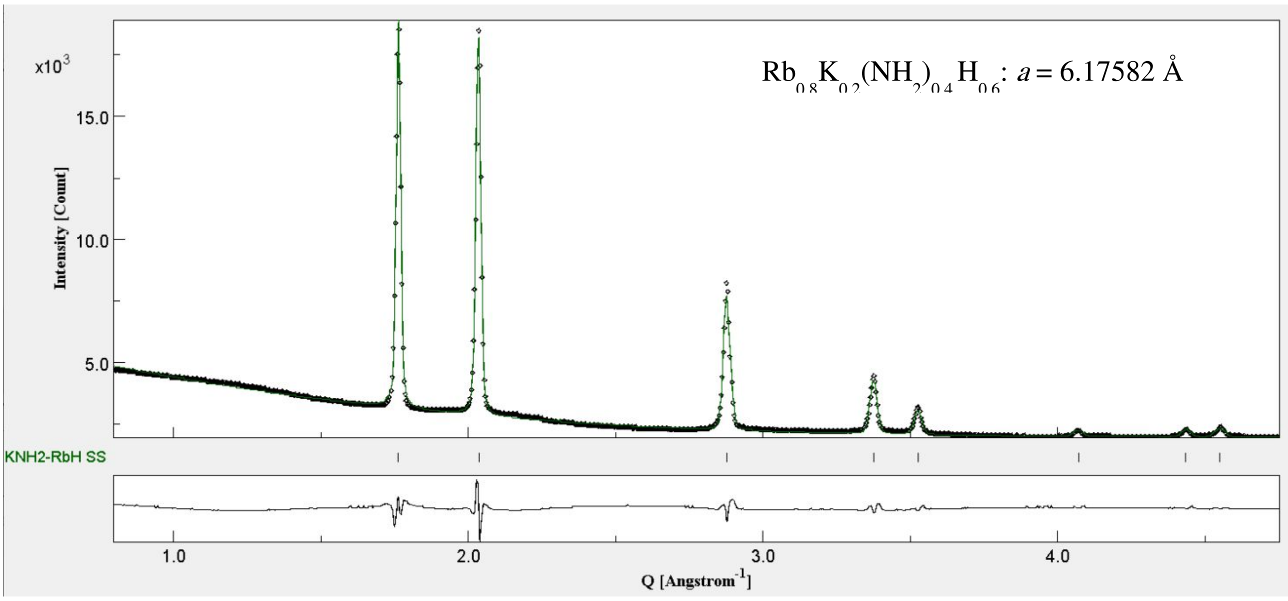

(h) $0.5KNH_2$+0.5RbH (sigma = 2.24, R_{wp} = 3.86 %, R_{exp} = 1.72 %)

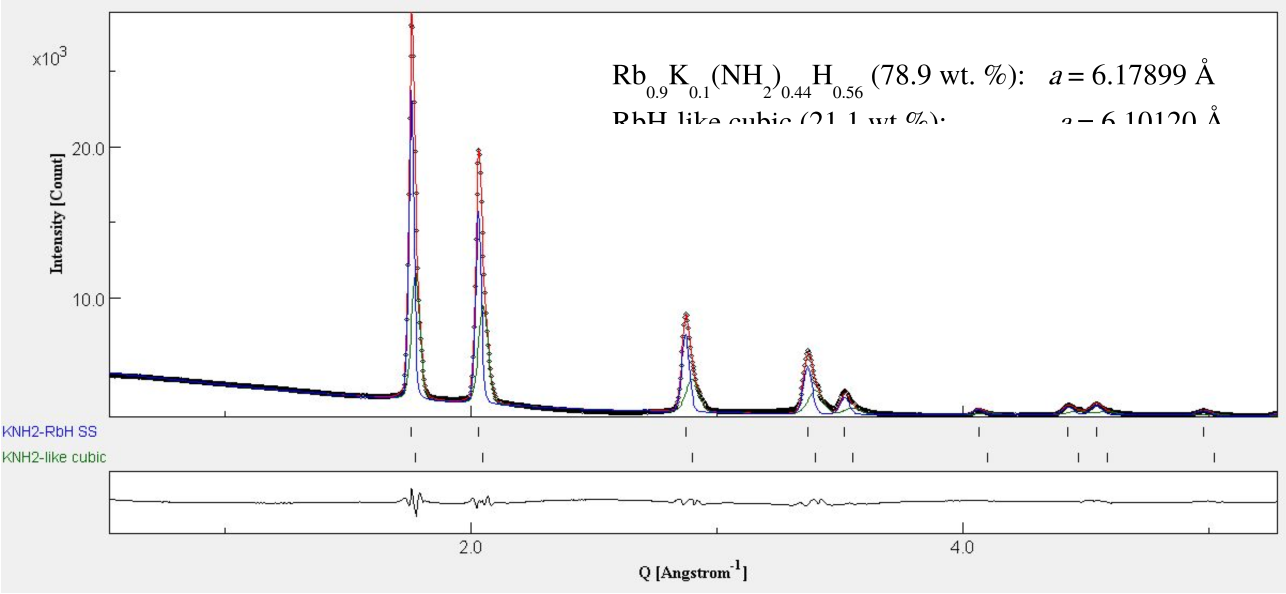

Figure S4. Rietveld refinement of *in-situ* SR-PXD data acquired at 270 ˚C for starting and mixed samples: (a) $RbNH_2$, (b) KH, (c) $0.7RbNH_2$+0.3KH, (d) $0.5RbNH_2$+0.5KH, (e) KNH_2, (f) RbH, (g) $0.7KNH_2$+0.3RbH and (h) $0.5KNH_2$+0.5RbH.

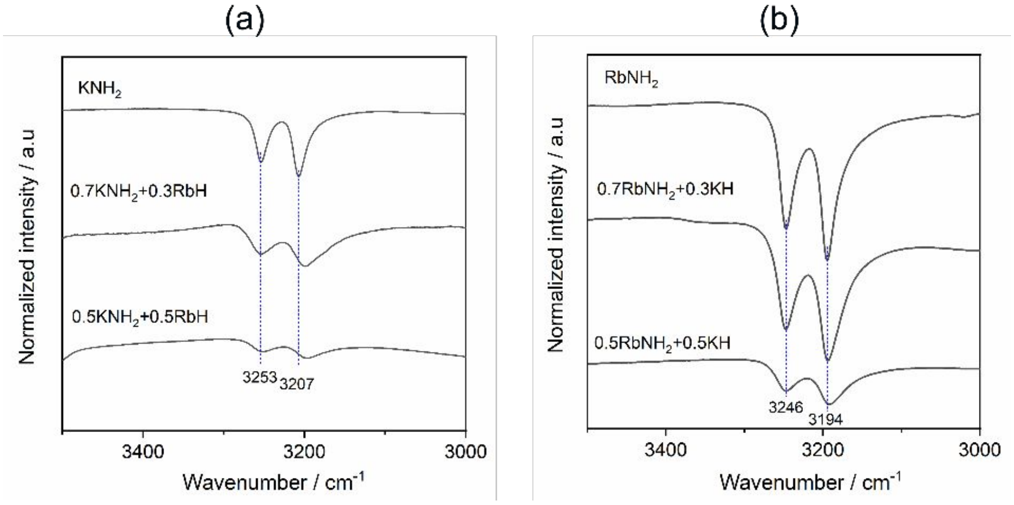

Figure S5. FT-IR of (a) $xKNH_2+(1-x)RbH$ and (b) $xRbNH_2+(1-x)KH$ samples.
